# The complete chloroplast genome sequence of *Xylosma congesta*

**DOI:** 10.1080/23802359.2019.1670746

**Published:** 2019-10-01

**Authors:** Yang Tian, Shuyu Liu, Yi Li, Jianguo Zhang, Zhaoshan Wang

**Affiliations:** State Key Laboratory of Tree Genetics and Breeding, Key Laboratory of Silviculture of the State Forestry Administration, Research Institute of Forestry, Chinese Academy of Forestry, Beijing, China

**Keywords:** *Xylosma congesta*, complete chloroplast genome, phylogenetic relationship

## Abstract

We sequenced and analyzed the complete chloroplast genome of *Xylosma congesta.* The chloroplast genome was 156,520 bp in size, containing a large single-copy (84,404 bp) and a small single-copy region (16,474 bp) separated by two inverted repeat regions of 27,821 bp each. A total of 130 genes were annotated, including 86 protein-coding genes, 36 tRNA genes, and 8 rRNA genes. The overall GC content of the chloroplast genome was 36.7%. The phylogenomic analysis strongly supported the close relationship between *X. congesta* and *Flacourtia indica*.

*Xylosma congesta* belongs to the genus *Xylosma* of the family Salicaceae. *Xylosma congesta* is an evergreen small tree or large shrub distributed in India, western China, Japan, and South Korea (Zmarzty 2010). It appears in plains, shrubs, and forest margins, and seeds can be extracted into oil (Xie et al. 2013). The material of *X. congesta* is solid and suitable for making furniture. In addition, its leaves and thorns can be used for medicinal treatment. Here, we have reported and analyzed the chloroplast genome of *X. congesta* to provide information for further study and utilization of this species.

Fresh leaves of *X. congesta* were collected from Anshun City, Guizhou Province, China (26°25'N, 105°92'E). The voucher specimen (*Xylcon002*) was laid in the herbarium of Forestry Research Institute of Chinese Academy of Forestry and the extracted DNA is stored in the −80 °C refrigerator of the Research Institute of Forestry. We extracted the total genomic DNA from leaves preserved in silica gel desiccant with modified CATB method (Doyle and Doyle 1987). Genome sequencing was performed on an Illumina HiSeq 2000 platform (Illumina, San Diego, CA). We used the software MITObim 1.8 (Hahn et al. 2013) to assemble chloroplast genomes. At the same time, *Flacourtia indica* (GenBank: MG262341) was used as a reference genome. We annotated the chloroplast genome with the software DOGMA (Wyman et al. 2004) and then corrected the results using Geneious 8.0.2 (Kearse et al. 2012) and Sequin 15.50 (http://www.ncbi.nlm.nih.gov/Sequin/).

The complete chloroplast genome of *X. congesta* (GenBank accession MK609964) has a circular molecular structure of 156,520 bp in length with a large single-copy (LSC) region of 84,404 bp, a small single-copy (SSC) region of 16,474 bp, separated by two inverted repeat (IR) regions of 27,821 bp each. The chloroplast genome contains 130 genes in total, consisting of 86 protein-coding genes (77 protein-coding gene species), 36 tRNA genes (29 tRNA species), and 8 rRNA genes (4 rRNA species). Most of these are single-copy genes, while 15 genes (6 PCGs genes, 4 rRNA genes, and 5 tRNA genes) are located in the IR regions. Overall GC content of chloroplast genome was 36.7% and in LSC, SSC, and IR regions were 34.6, 30.8, and 41.9%, respectively.

We used the complete chloroplast genomes sequence of *X. congesta* and 10 other related species of Salicaceae and *Hevea brasiliensis* as the outgroup to construct the phylogenetic tree. 12 chloroplast genome sequences were aligned with MAFFT (Katoh and Standley 2013), and then the maximum-likelihood tree was constructed by MEGA 7.0 (Kumar et al. 2016). The results confirmed that *X. congesta* was clustered with *Flacourtia indica* (Figure 1).

**Figure 1.**
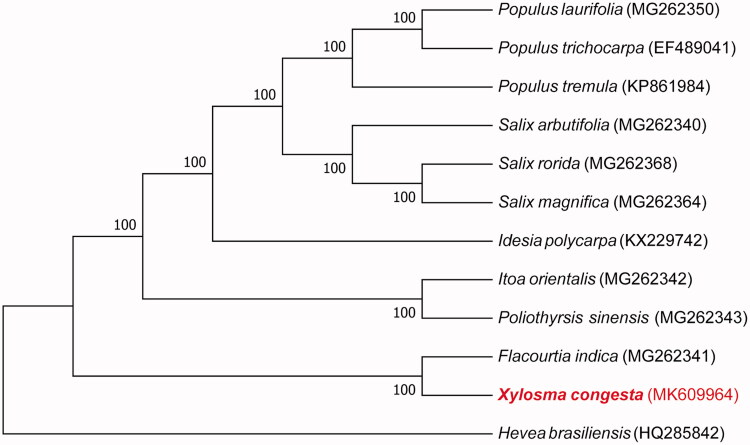
Maximum-likelihood phylogenetic tree for *X. congesta* based on 12 complete chloroplast genomes. The Genbank accession numbers are on the diagram.
